# IsoPlot: a database for comparison of mRNA isoforms in fruit fly and mosquitoes

**DOI:** 10.1093/database/bax069

**Published:** 2017-09-12

**Authors:** I-Man Ng, Jia-Hsin Huang, Shang-Chi Tsai, Huai-Kuang Tsai

**Affiliations:** 1 Institute of Information Science, Academia Sinica, 128 Academia Road, Section 2, Nankang, Taipei 115, Taiwan

## Abstract

Alternative splicing (AS), a mechanism by which different forms of mature messenger RNAs (mRNAs) are generated from the same gene, widely occurs in the metazoan genomes. Knowledge about isoform variants and abundance is crucial for understanding the functional context in the molecular diversity of the species. With increasing transcriptome data of model and non-model species, a database for visualization and comparison of AS events with up-to-date information is needed for further research. IsoPlot is a publicly available database with visualization tools for exploration of AS events, including three major species of mosquitoes, *Aedes aegypti*, *Anopheles gambiae*, and *Culex quinquefasciatus*, and fruit fly *Drosophila melanogaster*, the model insect species. IsoPlot includes not only 88,663 annotated transcripts but also 17,037 newly predicted transcripts from massive transcriptome data at different developmental stages of mosquitoes. The web interface enables users to explore the patterns and abundance of isoforms in different experimental conditions as well as cross-species sequence comparison of orthologous transcripts. IsoPlot provides a platform for researchers to access comprehensive information about AS events in mosquitoes and fruit fly. Our database is available on the web via an interactive user interface with an intuitive graphical design, which is applicable for the comparison of complex isoforms within or between species.

**Database URL**: http://isoplot.iis.sinica.edu.tw/

## Introduction

Gene splicing endows the metazoan genome with transcriptional diversity and complexity. Alternative splicing (AS), the selective removal and recombination of exons, is known to play a pivotal role in regulatory pathways from invertebrates to human (1). Through AS, a single gene is capable of generating various mRNA isoforms that encode proteins with different functions (2). The importance of AS lies in the evidence that >95% of multi-exon genes in human undergone AS (3, 4), and dysregulation of AS is prevalent to associate with many complex diseases (5, 6). Moreover, AS events and their abundance provide a crucial step for understanding the functional context of gene expression in different tissues and developmental stages of invertebrates and vertebrates (7, 8).

The construction of AS database can facilitate the identification, classification, and functional annotation of RNA splice variants. Despite human AS databases are continuously updated with newly published data (9–11), AS databases of the model insect species *Drosophila melanogaster* (12, 13) were out-of-date due to the lack of recently annotated splice isoforms in the latest version of the reference sequence of the *D. melanogaster* genome (Release 6) (14). Moreover, the AS data are complex and the visualizations in the existing AS databases are often not interactive for exploration. On the other hand, AS databases are mostly unavailable for non-model organisms.

Mosquitoes are vectors for many important diseases such as malaria, dengue, Zika, and yellow fever that are global scourges and cause millions of deaths worldwide annually (15). The characterization of alternative splicing in genes can facilitate our understanding on mosquito fundamental physiology and behavior for different control strategies. For instance, the genes involved in immune response and olfaction can provide new targets for strategies aimed at making refractory mosquitoes (16) and disruption of host-seeking or oviposition behaviors (17, 18). However, annotation of alternatively spliced isoforms in mosquitoes is far from comprehensive in current platforms such as VectorBase (19), which seldom devotes to the exploitation of splice isoforms. The growing RNA-Seq data for several mosquito species at different developmental stages and conditions have put the detection of mRNA isoforms in practice. Consequently, isoform identification and quantification from existing RNA-Seq data are essential for understanding fundamental biological processes and revealing crucial factors in the control of vector-borne diseases.

We therefore have developed IsoPlot, a web-based database with an interactive visualization of AS events. Our database comprises three species of mosquitoes, *Aedes aegypti*, *Anopheles gambiae*, and *Culex quinquefasciatus*, that are major vectors for diseases and one model species *D. melanogaster*, providing more well-annotated transcripts for isoform comparison. For mosquitoes, annotation files and large-scale RNA-Seq experimental data were collected and processed to predict new transcripts and to calculate transcript-level expression. Notably, scientists can apply IsoPlot to (i) identify distinctive events of splicing variations under different experimental conditions and (ii) to compare splice isoforms of orthologous genes in neighboring species through pairwise sequence alignment. IsoPlot is a useful platform to biologists for aiding mosquito vector research, and facilitating improvements in public health.

## Database construction

### Database architecture

**Figure 1.**
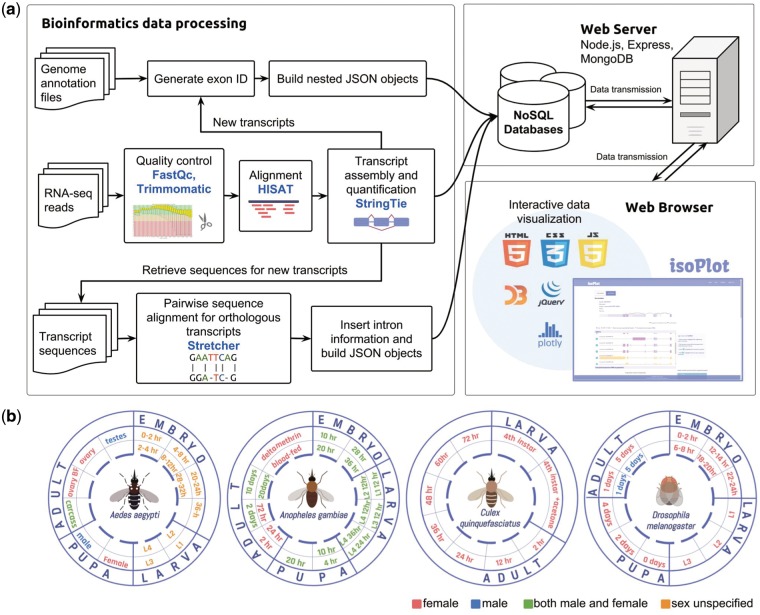
Data collection, processing and database scheme for IsoPlot. (**A**) The pipeline for isoform visualization, transcript-level expression analysis of RNA-Seq experiments, identification of new transcripts and sequence comparison. (**B**) Overview of RNA-Seq data generated for different developmental stages and conditions in four species.

The backend of our database was built with Node.js, Express and MongoDB framework to create a smoother user experience and to ease the technical maintenance of the system (20) (Figure 1A). Both the client and server side were written in Javascript, with the application of D3.js (21), Jquery (22) and Ploty (23) in developing SVG (Scalable Vector Graphics) elements and interface interactions. These javascript graphing libraries greatly enrich the graphic representations in IsoPlot with built-in animation. Our database encompasses RNA-Seq experiments of four insect species from different developmental time points, sex, and under the application of insecticides (Figure 1B). All the genome annotation files and alignment results were built into nested objects using JSON (JavaScript Object Notation) for our NoSQL approaches. The new genome annotation files in JSON and GTF (General Transfer Format) can be downloaded directly from our website.

### Species and genome version

**Figure 2.**
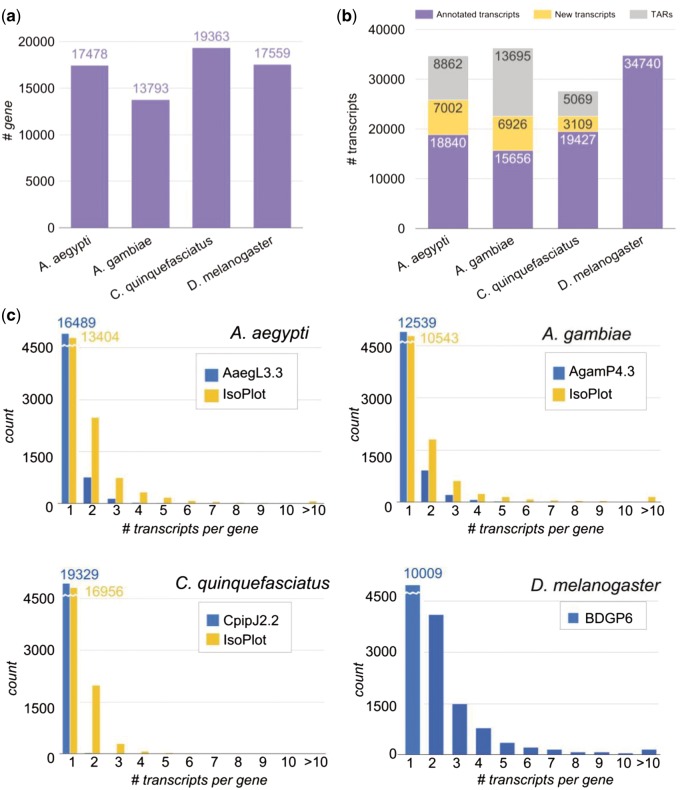
Number of genes and transcripts in the four species. (**A**) Number of genes in each species. (**B**) Number of annotated transcripts, new transcripts, and transcriptionally active regions (TARs) in the species. (**C**) Distribution of isoform number per gene in the four species. Blue bars denote the information in genome annotation files in each species, and yellow bars denote the information in the IsoPlot for the three mosquitoes.

The genome version of the three mosquito species, *A. aegypti*, *A. gambiae*, and *C. quinquefasciatus*, and the fruit fly *D. melanogaster* are AaegL3, AgamP4, CpipJ2, and BDGP6 respectively. Reference genomes and annotation files of mosquitoes were downloaded from VectorBase (19) and that of fruit fly from Ensembl (24). The number of annotated genes is shown in Figure 2A. For comparison of homologous genes among the four species, we first retrieved their orthologous relationship from OrthoDB v9 (25), and then employed the key-value pair (KVP) model in setting up an ortholog database for effective search query.

### RNA-seq processing workflow

As opposed to the abundant data in the model species *D. melanogaster*, we discovered that there are scarcely isoform variations in mosquito genome annotation files (Figure 2B and C). To improve this situation, we collected and analyzed large amounts of raw RNA-Seq experiments in different developmental stages of the mosquitoes, to enlarge our isoform variety. All the experimental data were collected from ArrayExpress and NCBI (National Center for Biotechnology Information) for the same strain of each mosquito species. In total, the sources contain 101 samples with over 13 billion sequence reads from four different developmental stages of insects (Supplementary Table S1).

The processing pipeline for RNA-Seq data is shown in (Figure 1A). Raw sequence data were investigated for quality using FastQC (26), then we used Trimmomatic (27) to remove low quality reads, and trimmed adapters with adapter files provided by Trimmomatic. We performed read alignment with HISAT2 (28). Transcript assembly and quantification were carried out by StringTie (28) to predict new transcripts and to obtain transcript-level expression for the mosquitoes. Since the transcript isoforms in *D. melanogaster* have been thoroughly annotated from modENCODE project (29, 30) and updated in the current version of *Drosophila* reference genome (Release 6) (14), we performed an alternate gene expression analysis workflow provided by StringTie which generates no novel isoforms. We ran the tools using default parameters. The output predicted a total of 66 864 new transcripts in the three mosquito species. In order to reduce false positives that might come from products of non-specific background transcription or mapping artefact, we first removed the unannotated genes and their corresponding transcripts. Second, because it has been shown that a minimum FPKM (fragments of transcript sequence per kilobase million) values of 0.1 could reflect active transcription (31), we selected the predicted transcripts having FPKM value > 0.1 in at least two samples per species. In addition, the predicted transcripts that contained any exon located outside the annotated gene region were considered as transcriptionally active regions (TARs), and removed from further expression analysis. Ultimately, we discovered 17 037 novel transcripts and 27 626 TARs in the three mosquitoes (Figure 2B). The newly predicted transcripts largely increased the numbers of isoforms in the three mosquito species (Figure 2C, yellow bars).

### Sequence identity of transcripts

**Figure 3.**
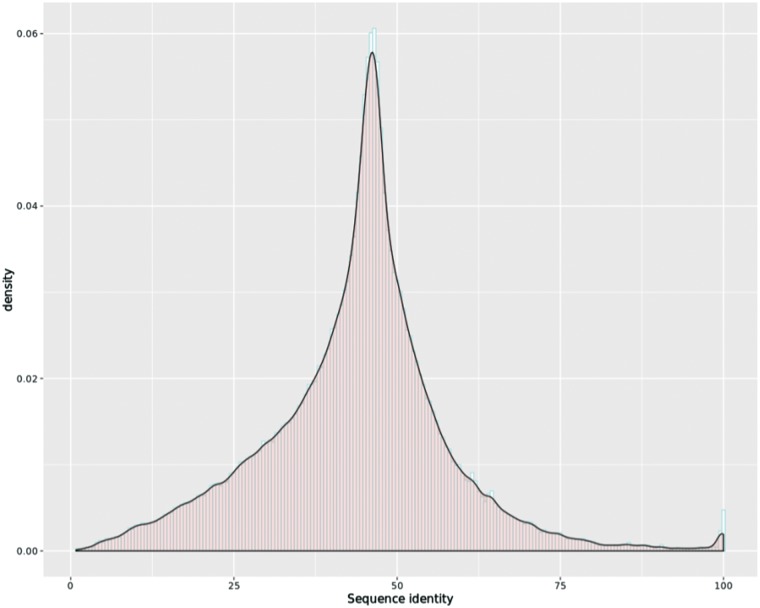
Histogram overlaid with kernel density curve for sequence identity of the 439 215 orthologous transcript pairs, with mean identity 43.85 and third quartile above 50.70.

To attain cross-species comparison of the orthologous transcripts, we first collected the genomic region of all the transcripts from our new annotation files and used samtools (32) to extract their sequences from their genome FASTA files. Next, we applied EMBOSS Stretcher (33) to calculate an optimal global sequence alignment for all the orthologous transcript pairs, including the newly assembled transcripts. The distribution of sequence identity of the 439 215 orthologous transcript pairs is shown in Figure 3, with mean identity 43.85 and third quartile above 50.70. The default Stretcher output was combined with intron information from the genome annotation files for visualization.

## Utility and discussion

**Figure 4.**
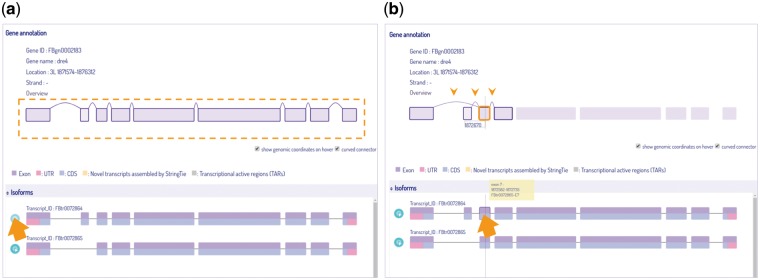
Isoform visualization in IsoPlot. Hover effects allow users to (**A**) highlight isoform structure of connected exons or (**B**) juxtaposition events of an exon (three arrow heads) for information and comparison. A crosshair line indicates the current genome position.

We include a phylogenetic tree in the query page for user to choose reference species at a particular ortholog node, and generate results for either a single gene or a group of selected orthologs for cross-species comparison. Our viewer provides basic information and depicts all isoform variations of the gene, with a link connecting to its reference database where detailed information is offered. The three main components in IsoPlot are isoform visualization, transcript-level expression analysis, and cross-species isoform sequence comparison. Isoforms are scaled by original genome coordinates including introns, with a cursor that indicates the current genomic coordinate. Hovering and animated sorting effects are also utilized to make IsoPlot interactive and alive (Figure 4). Users can rearrange isoform order or highlight exon connections through hovering and clicking.

**Figure 5.**
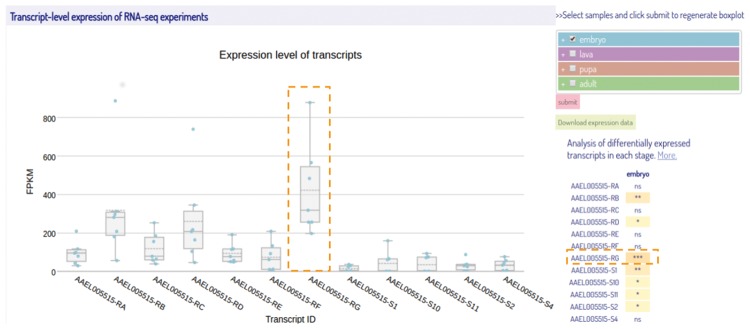
A screenshot of the transcript-level expression view of the selected RNA-Seq samples. The box-plots represent the expression levels of the isoforms from different samples during embryonic stage. On the right of the box-plot is a multi-select tree for sample selection and a summary table of statistical results. The samples at four different developmental stages are represented with different colors same as shown in the multi-select tree. The transcript highlighted by a dashed box is the most differentially expressed transcript among all others in the embryonic stage with the significance determined by Wilcoxon rank sum test. (***P-value < 0.001).

Isoform expressions are represented as FPKM values of transcripts using box plots (Figure 5). Here, we applied Plotly (23), a javascript graphing library, in creating an interactive boxplot. There is a list for users to select their samples and stages of interest and the plot is updated dynamically. Four developmental stages of insect (embryo, larva, pupa, and adult) are grouped and represented in different colors. In order to compare the expression levels of each isoform during a particular developmental stage, a two-sided Wilcoxon rank sum Test (34) was performed to examine whether an individual transcript was differentially expressed against all other transcripts. Right to the boxplot is a summary table that represents the significant *P*-values for each transcript using a real-time javascript implementation (35). Users can also download the expression data in FPKM values for the transcripts of interest from IsoPlot for further analyses. It should be noted that the transcriptome data were collected from different sources and sequencing platforms, with various sequencing depth and library preparation. The heterogeneity of the original transcriptome data might potentially affect expression analysis across species although we have conducted an unambiguous pipeline.

**Figure 6.**
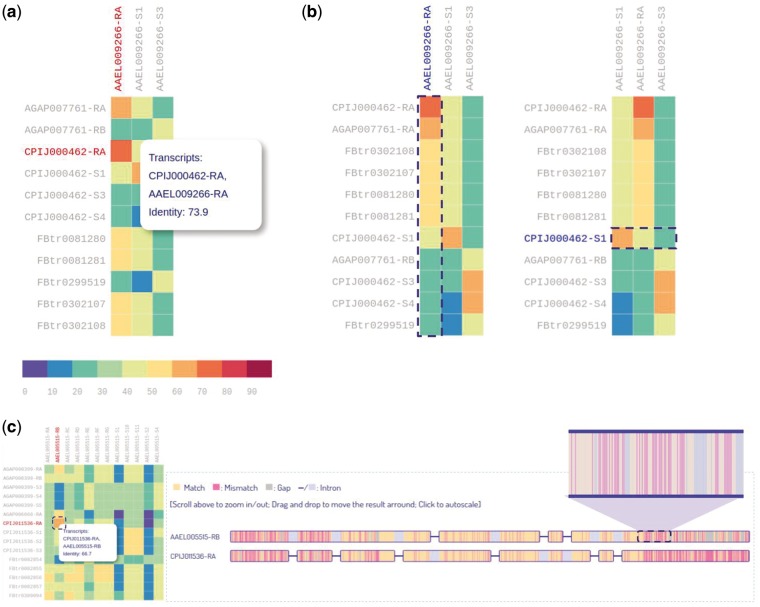
Heatmaps for sequence identity of orthologous transcripts. (**A**) Users can hover over a cell to display identity score. (**B**) Users can interactively sort the columns or rows of the heatmap by identity score. (**C**) Zoomable visualization of alignment result for two transcripts.

For isoform sequence comparison, we provided a heatmap with each colored cell representing the sequence identity of two orthologous transcripts (Figure 6A). The heatmap is interactive and can be dynamically sorted by either ascending or descending order on both columns and rows (Figure 6B). In this way users can easily target the transcript with the highest identity among its orthologs. Moreover, by clicking a heatmap cell, the alignment result of the transcript pair can be visualized or downloaded from our database. The visualization of alignment result for two transcripts is shown in Figure 6C. The visualization is pannable and zoomable, allowing users to gain insight into the alignment results readily. A total of 439 215 alignment results can be queried and visualized from IsoPlot.

The visualization tools in IsoPlot enable scientists to explore the patterns of different transcript isoforms in other species. We provide the source code for JavaScript implementation of this workflow in a Github repository (https://github.com/mikekd106/isoPlot). In the future, we plan to improve IsoPlot by including more transcriptome data so that IsoPlot is available for the exploitation of transcriptomic diversity with more species/strains, and incorporating additional features based on requests from user feedback.

## Conclusions

We have collected large amounts of transcriptome data at different stages and conditions of three mosquito species and *D. melanogaster*. In addition to the most up-to-date information of annotated isoforms in the four insect species, we also identified 17 037 novel transcripts in the three mosquito species. Through IsoPlot database, users can browse and compare the splicing variants for transcript-level expression within species and for sequence comparison between species with interactive visualization tools. Moreover, the availability of the complete set of splicing transcripts in the analyzed species will improve the comparative and evolutionary analyses of AS events among different Diptera species. In conclusion, we believe that IsoPlot will serve as a useful platform for exploration of AS events and applications on the mosquito control in future.

## Availability of data and materials

IsoPlot is based on HTML5/SVG technology and is freely available at http://isoplot.iis.sinica.edu.tw/.

## Supplementary data

Supplementary data are available at *Database* Online.

## Supplementary Material

Supplementary Data
